# Supplementary material for: The Rm1 and Rm2 Resistance Genes to Green Peach Aphid (Myzus persicae) Encode the Same TNL Proteins in Peach (Prunus persica L.)
Source: Genes (Basel). 2022 Aug 20;13(8):1489. doi: 10.3390/genes13081489 (PMC9408794; doi:10.3390/genes13081489)
Supplement: Supplementary file 1 [file genes-13-01489-s001.zip › genes-1839843-supplementary/Supplemental_Figures.pdf]

Supplemental figures for the article :

Figure S1: FGENESH 2.6 Prediction of potential genes in the *Prunus persica* genomic DNA sequence RM1bac

Figure S2: Mapping of the WFP RNAseq Long-read and the WFP, Rubira, Montclar NGS short-reads onto the Rm1bac sequence at the position of the *TNL1* gene.

Figure S3: TNL1 protein sequence with annotations of the TIR, NBS, NLL, LRR, PL domain motifs. The exon starts are indicated with a triangle

Figure S4: TNL2 protein sequence with annotations of the TIR, NBS, NLL, LRR, PL domain motifs.

Figure S1: **FGENESH 2.6 Prediction of potential genes in the *Prunus persica* genomic DNA sequence RM1bac**

Seq name: RM1Bacs_ Length of sequence: 517362

Number of predicted genes 116: in +chain 59, in -chain 57.

Number of predicted exons 488: in +chain 236, in -chain 252.

Positions of predicted genes and exons: Variant 1 from 1, Score:9511.051563

CDSf CDSi CDSl CDSo PolA TSS

CDSf—First (starting with start codon);CDSi—internal (internal exon), CDSl—last coding segment (ending with stop codon); CDSo: coding sequence, solo—predicted gene with a single exon; PolA—polyadenylation signal sequence (AATAAA), and TSS—position of transcription start (TATA-box position and score)

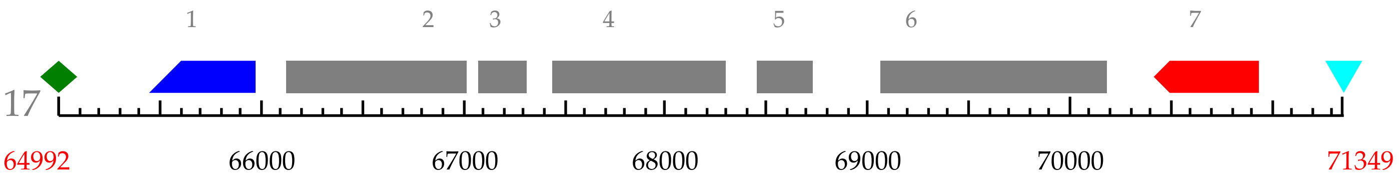

17 -	PolA	64992		1.88			
17 -	1 CDSl	65439 -	65966	25.83	65439 -	65966	528
17 -	2 CDSi	66117 -	67010	45.21	66117 -	67010	894
17 -	3 CDSi	67067 -	67306	0.41	67067 -	67306	240
17 -	4 CDSi	67434 -	68291	25.76	67434 -	68291	858
17 -	5 CDSi	68446 -	68721	10.84	68446 -	68721	276
17 -	6 CDSi	69058 -	70177	70.11	69058 -	70176	1119
17 -	7 CDSf	70409 -	70929	45.90	70411 -	70929	519
17 -	TSS	71349		-4.12			

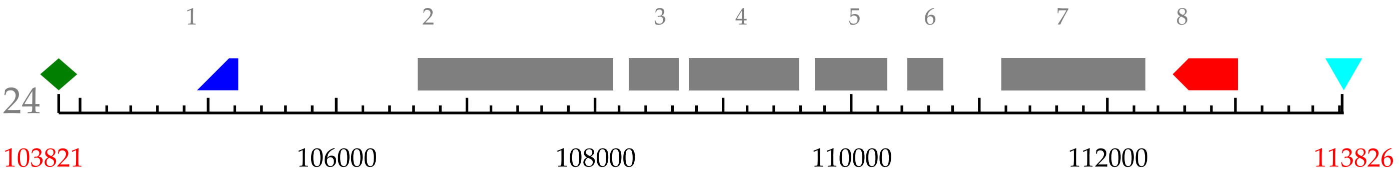

24 -	PolA	103821		-2.92			
24 -	1 CDSl	104898 -	105219	8.27	104898 -	105218	321
24 -	2 CDSi	106617 -	108136	160.54	106619 -	108136	1518
24 -	3 CDSi	108259 -	108647	15.09	108259 -	108645	387
24 -	4 CDSi	108727 -	109585	37.92	108728 -	109585	858
24 -	5 CDSi	109710 -	110273	13.36	109710 -	110273	564
24 -	6 CDSi	110428 -	110706	16.25	110428 -	110706	279
24 -	7 CDSi	111162 -	112281	54.03	111162 -	112280	1119
24 -	8 CDSf	112494 -	113002	37.95	112496 -	113002	507
24 -	TSS	113826		-1.32			

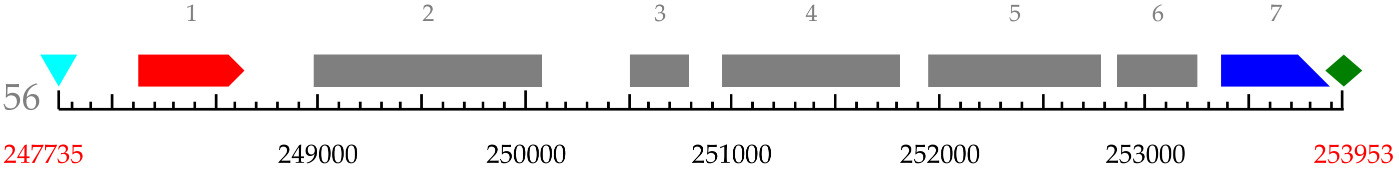

56 +	TSS	247735		-3.60			
56 +	1 CDSf	248120 -	248634	42.28	248120 -	248632	513
56 +	2 CDSi	248969 -	250073	70.06	248970 -	250073	1104
56 +	3 CDSi	250499 -	250786	10.16	250499 -	250786	288
56 +	4 CDSi	250946 -	251803	21.16	250946 -	251803	858
56 +	5 CDSi	251942 -	252776	32.57	251942 -	252775	834
56 +	6 CDSi	252856 -	253244	14.26	252858 -	253244	387
56 +	7 CDSl	253359 -	253886	29.20	253359 -	253886	528
56 +	PolA	253953		-4.22			

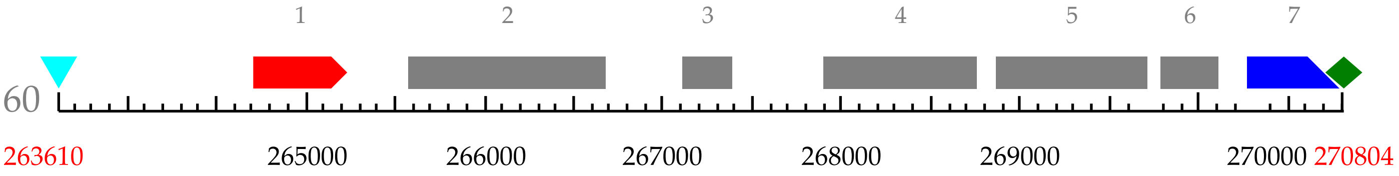

60 +	TSS	263610		-2.22			
60 +	1 CDSf	264699 -	265225	42.87	264699 -	265223	525
60 +	2 CDSi	265568 -	266672	57.33	265569 -	266672	1104
60 +	3 CDSi	267102 -	267380	7.03	267102 -	267380	279
60 +	4 CDSi	267891 -	268748	25.31	267891 -	268748	858
60 +	5 CDSi	268856 -	269702	39.95	268856 -	269701	846
60 +	6 CDSi	269779 -	270101	15.41	269781 -	270101	321

60 +	7 CDSl	270262 -	270780	22.99	270262 -	270780	519
60 +	PolA	270804		-4.92			

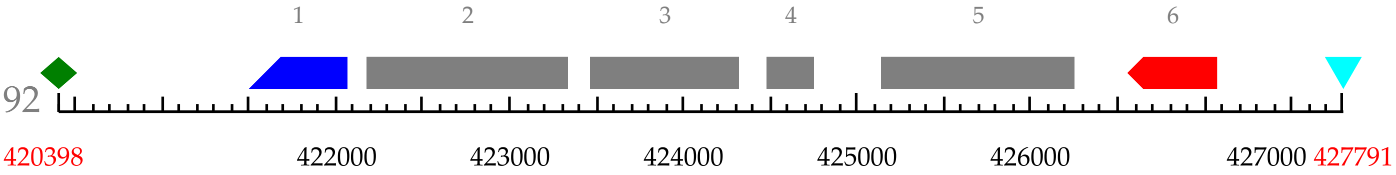

92 -	PolA	420398		1.88			
92 -	1 CDSl	421491 -	422060	28.06	421491 -	422060	570
92 -	2 CDSi	422171 -	423328	56.99	422171 -	423328	1158
92 -	3 CDSi	423456 -	424313	31.34	423456 -	424313	858
92 -	4 CDSi	424473 -	424745	7.33	424473 -	424745	273
92 -	5 CDSi	425131 -	426244	54.70	425131 -	426243	1113
92 -	6 CDSf	426548 -	427065	39.62	426550 -	427065	516
92 -	TSS	427791		-1.61			

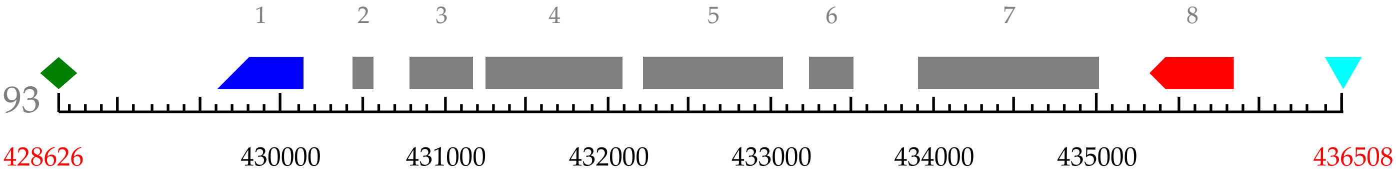

93 -	PolA	428626		1.88			
93 -	1 CDSl	429598 -	430128	23.77	429598 -	430128	531
93 -	2 CDSi	430429 -	430557	0.57	430429 -	430557	129
93 -	3 CDSi	430778 -	431166	13.66	430778 -	431164	387
93 -	4 CDSi	431246 -	432086	43.70	431247 -	432086	840
93 -	5 CDSi	432212 -	433069	26.84	432212 -	433069	858
93 -	6 CDSi	433229 -	433501	14.25	433229 -	433501	273
93 -	7 CDSi	433900 -	435010	62.88	433900 -	435009	1110
93 -	8 CDSf	435319 -	435836	38.51	435321 -	435836	516
93 -	TSS	436508		-0.02			

Figure S2: Mapping of the WFP RNAseq Long-read and the WFP, Rubira, Montclar NGS short-reads onto the Rm1bac sequence at the position of the *TNL1* gene.

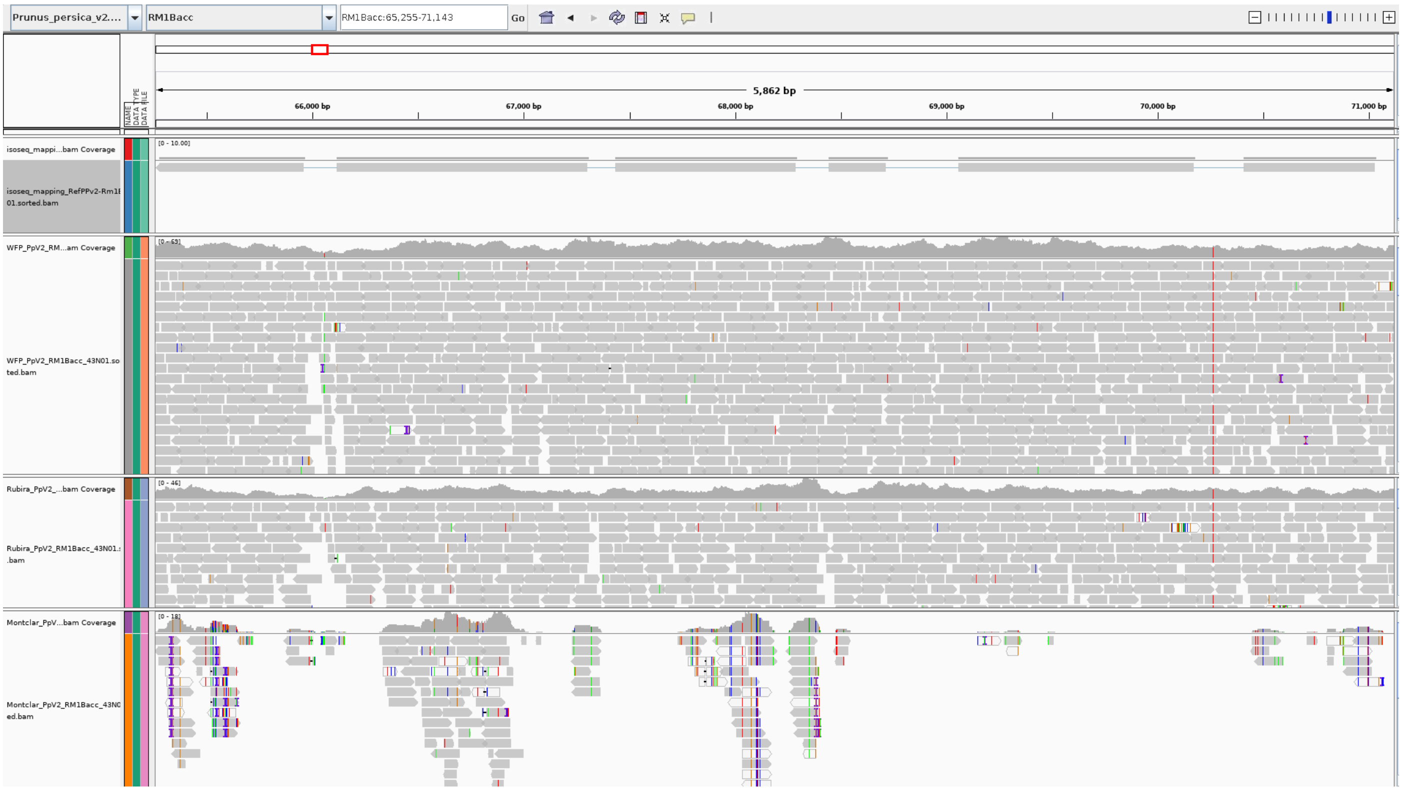

Figure S3: TNL1 protein sequence with annotations of the TIR, NBS, NLL, LRR, PL domain motifs. The exon starts are indicated with a triangle.

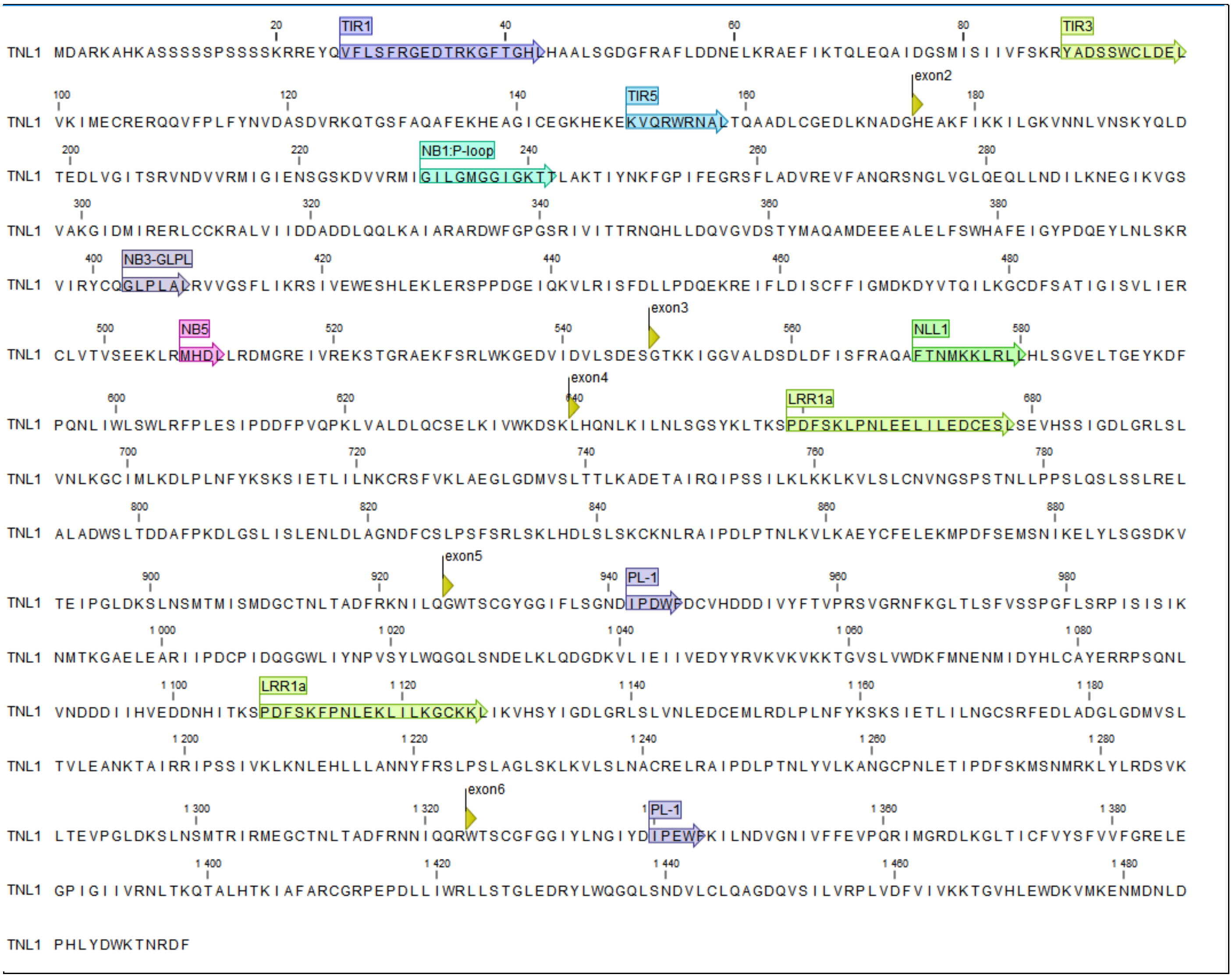

Figure S4: TNL2 protein sequence with annotations of the TIR, NBS, NLL, LRR, PL domain motifs.

```
TNL 2 MGDGTSIEGPSSRTWKYQVFLSFRGEDTRKGFTGHLHAALSDAGIRAFLDDNELERTEYIKNQLEQAIHGSMISIIVFSKRYADSSWCLDELVKIMECRERLGQKVFPLFYNVDASDVRNQTGS
TNL 2 FAQAFEKHEAGICEGKHEKEKVQRWKTALKNAADMCGEDRKNADNGHEAKFIKKVLGEVDKQLSSKYQLDIENLVGITSRLKVLSNHLDIENSGSKDVVRMIGILGMGGIGKTTLATTIYNKFE
TNL 2 RIFEGRSFLANVREVIAHQASTGLVGLQEQLLNDILKSQGIKVDSVAKGIDMIRARLCCKRALVIIDDADDLQQLKAIAGARDWFGHGSRIIITTRNKHLLVQVGVDSTYMAQEMCKEEALELF
TNL 2 SLHAFKRDYPDQEYLDLSKRVIHYCQGLPLALRVVGSFLIKRSIAEWESHLEKLERSPDGDIQKILRISFDGLPDQEKREIFLDISCFFIGMDKDYVTQILKGCGFAQPIGISVLIERCLVTVS
TNL 2 EENKLMMHDLLRDMGREIVYENAQGHCEKFSRLWKREDVTDVLSDESGTEEIEGVALDLPWSDKASFSAEAFTNMKKLRLLDLSNVELTGEYKDFPKKLIFLRWPGFPLESIPDDFPMQPKLVA
TNL 2 LDLHFSKLKIVWKDCKLHQNLKIINLSYSLELTKLPDFSKLPNLEELILEGCESLSHVHPSIGDLGRLSLVNLKGCKKLRDLPLNFYKSKSLTTLKADETAIRQLPNLSHLSQLQECLSLNMCQ
TNL 2 KLHAIPDLPTNLKVLEANFCLGLEKIPDFSEMSNIRTLCLSDSDKLTEIPGLDKSLNSMTRIHMDNCTNLTADFRKNILQGWTSCGYGGIFLSGNDIPDWFDCVHDDDIVYFTVPPSVGRNLKG
TNL 2 LTLSFVYSSFPLFSGHISIKNMTEGTELDARIIPDSPTRDWDTYYVWQGQLSNDELKLQDGDKILIEIITEDKYGFGNPEVKKTGVSLVWDKFMNENMIDYHLCAYERRPSQNLVYVDDIIHVE
TNL 2 YHYCIRKLPDFSRFPNLEKLILKGCGNLFKVHSSIGDLGRLSLVNLEDCKMLRDLPLNFYKSKSIETLLLNGCSGFQNLADGLGDMVSLTILKADNTRIRQIPSSIVKLKKLRILSLSGCWLTE
TNL 2 DAIPKDLCSLISLEDLLLEANSFRSLPSLAGLSKLKVLCVNACKFLRAIPDLPTNLYVLKANGCPKLETIPDFSKMSNMRELYLSDSFKLTEVPGLDKSLNSMTRIHMEGCTNLTADFRNNILQ
TNL 2 RWTSCGFGGIYLNGIYDIPEWFELDDDVDNIVFFEVPQRIMGRDLKGLTICFVYSSDRRKLEDSEGPIDIIVRNLAKQTALHTKIAFASIKSSSEPEEPEDDSSEPKDASSEPEDDSSEPEEPE
TNL 2 DDSSEPEDDSSEPKDDSSEPEDDSSEPEDDSSEPKDASSEPEDDSSEPEDDSSEPEDDSSEPKDDSSEPEDDSSEPEEPEDDSSEPEDDSSEHEDDSIEPEDDSSEPEDDSIEHEDD
TNL 2 SSEPEEPKDDYLSPGQLSNESKDHYLWQGQLSNDVLRLQGGDKVTILVRPLVDFVRVKKTGVHLEWDKLMKENMDNPDPHLYDLETNRDFLAVKPYLDFLGGTDDEARPSHDASVPGDQVSAID
TNL 2 EESAVEDDPLGHGYLRTRLSLSIMENLDFSGGADDGAGRRHGAFVRTNQSSAVDKQLERDKVMKENMDNLDPHLYDLETNQDFSGGVDDAFFRGSVIHEQLAVEPYLGFLGGTDNEAGRSHDAF
TNL 2 VGNNQSSAVEGCFAHLKSFLFNLFFNCFSHPR
```
